# Supplementary material for: Unveiling the Genetic Mechanism of Meat Color in Pigs through GWAS, Multi-Tissue, and Single-Cell Transcriptome Signatures Exploration
Source: Int J Mol Sci. 2024 Mar 26;25(7):3682. doi: 10.3390/ijms25073682 (PMC11012088; doi:10.3390/ijms25073682)
Supplement: Supplementary file 1 [file ijms-25-03682-s001.zip › Supplementary Figure.pdf]

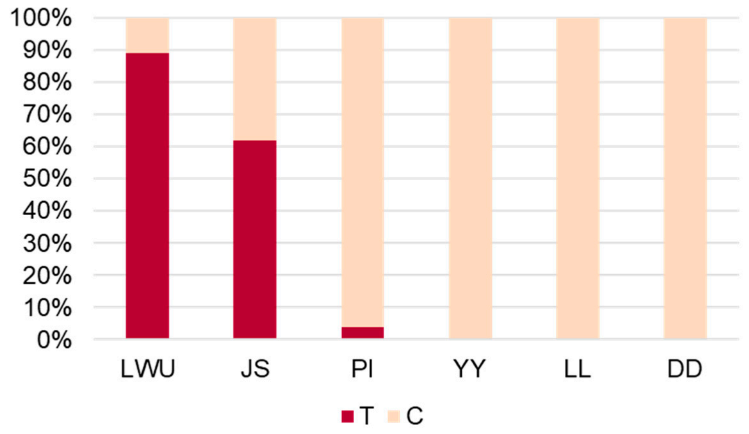

Figure S1. The allele frequency of the promising SNP in pigs with different meat color characteristics

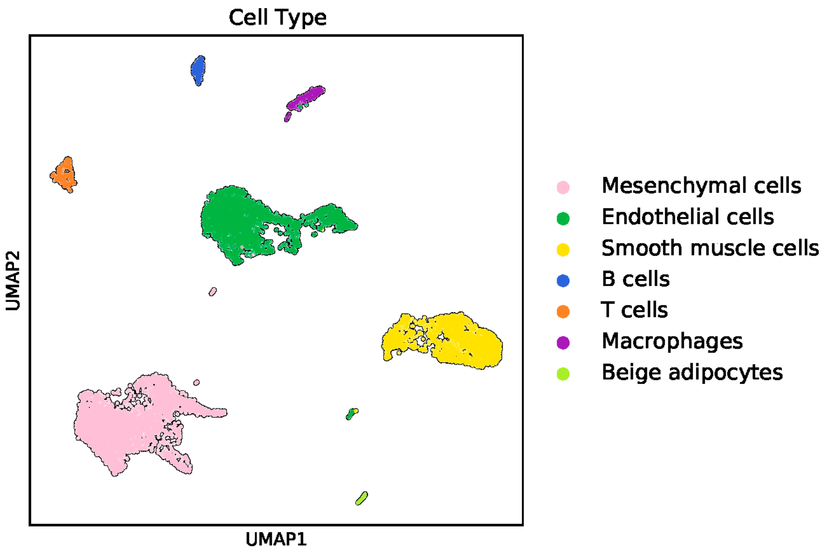

Figure S2. The uniform manifold approximation and projection (UMAP) of scRNA-seq data from adipose tissue

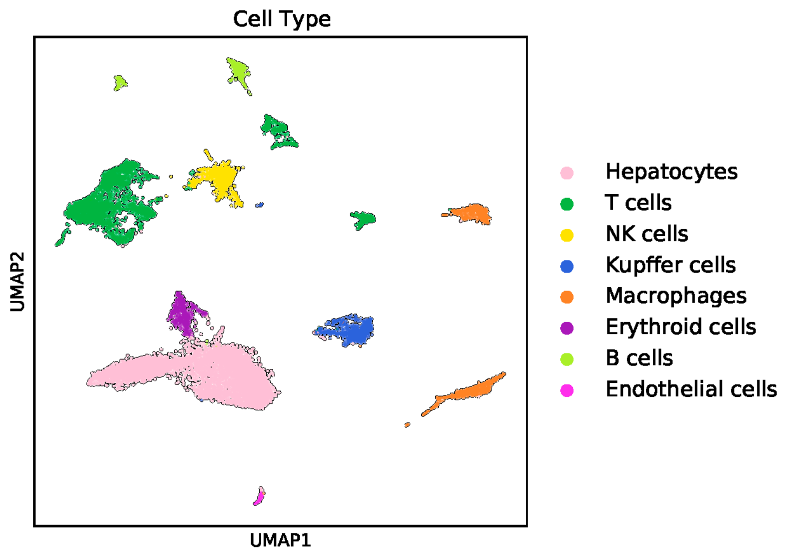

Figure S3. The uniform manifold approximation and projection (UMAP) of scRNA-seq data from liver tissue

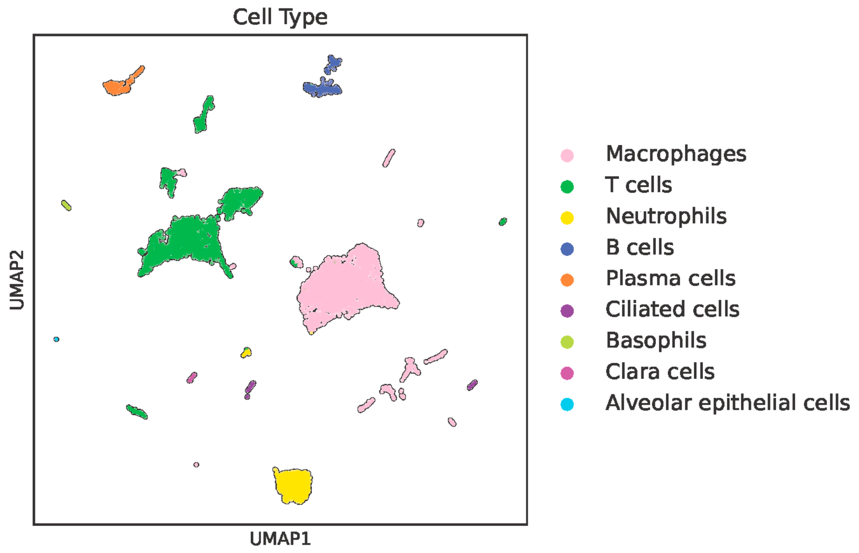

Figure S4. The uniform manifold approximation and projection (UMAP) of scRNA-seq data from lung tissue

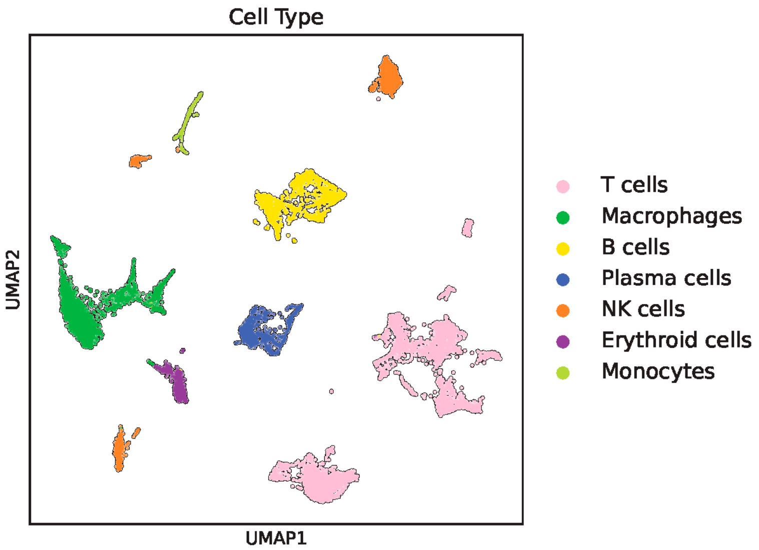

Figure S5. The uniform manifold approximation and projection (UMAP) of scRNA-seq data from spleen tissue
